# In vivo assessment of the antimalarial activity and acute oral toxicity of an ethanolic seed extract of *Spondias pinnata* (L.f.) Kurz

**DOI:** 10.1186/s12906-022-03546-9

**Published:** 2022-03-16

**Authors:** Prapaporn Chaniad, Arisara Phuwajaroanpong, Walaiporn Plirat, Tachpon Techarang, Arnon Chukaew, Chuchard Punsawad

**Affiliations:** 1 Department of Medical Science, School of Medicine, Walailak University, Nakhon Si Thammarat, 80160 Thailand; 2 Research Center in Tropical Pathobiology, Walailak University, Nakhon Si Thammarat, 80160 Thailand; 3 Chemistry Department, Faculty of Science and Technology, Suratthani Rajabhat University, Surat Thani, 84100 Thailand

**Keywords:** Antimalarial activity, *Spondias pinnata*, Toxicity, *Plasmodium berghei*, Malaria

## Abstract

**Background:**

In response to the persistent problem of malaria resistance, medicinal herbal plants can be used as a source of potential novel antimalarial agents. Therefore, the aim of this study was to evaluate the in vivo antimalarial activity and toxicity of an ethanolic seed extract of *Spondias pinnata* (L.f.) Kurz (*S. pinnata*).

**Methods:**

Qualitative phytochemical screening of the extract was performed using standard procedures, and the constituents were determined by gas chromatography–mass spectrometry (GC–MS). The in vivo antimalarial activity was assessed against the *Plasmodium berghei* ANKA strain in mice based on 4-day suppressive, curative and prophylactic tests. In addition, the acute toxicity of the extract was evaluated after oral administration of a single dose of 2,000 mg/kg body weight.

**Results:**

Phytochemical screening tests on the ethanolic *S. pinnata* seed extract revealed the presence of terpenoids, tannins, and coumarins. GC–MS analysis of the extract led to the identification of twenty-nine phytochemical compounds, including oleic acid amide, β-sitosterol, linoleic acid, oleic acid, protocatechuic acid, syringic acid and gallic acid. The results of the 4-day suppressive test revealed that mice treated with 250, 500, 600 and 800 mg/kg doses of the ethanolic *S. pinnata* seed extract showed significant parasitemia suppression in a dose-dependent manner, with 22.94, 49.01, 60.67 and 66.82% suppression, respectively, compared to that of the negative control group. All the doses of the ethanolic seed extract significantly suppressed parasitemia (*P* < 0.05) during the curative activity test and prolonged the mean survival time compared to those of the negative control group. However, the ethanolic seed extract displayed lower curative and prophylactic activities than the standard drug artesunate. In addition, the ethanolic seed extract showed no signs of toxicity in mice at a dose of 2,000 mg/kg body weight.

**Conclusion:**

The *S. pinnata* seed extract contains various phytochemical compounds with important medicinal properties. The extract showed a significant suppression of parasitemia in a dose-dependent manner, prolonged the mean survival time and exhibited significant curative and prophylactic activities. The overall results of this study demonstrated that the *S. pinnata* seed extract possessed promising in vivo antimalarial activity against *P. berghei* ANKA, with no toxicity. The findings from the present study provide scientific evidence supporting the use of *S. pinnata* seeds in the development of new drugs for malaria treatment. Additional studies are needed to isolate and identify the active compounds as well as to understand the mechanism of inhibition.

## Background

Currently, malaria remains a serious public health problem in several regions, especially tropical and subtropical zones [1–3], because it is a primary cause of morbidity and mortality. According to the World Health Organization (WHO) report in 2020 [4], there are approximately 229 million cases of malaria worldwide and 0.5 million deaths per year [5, 6]. There are reports of *Plasmodium* strains resistant to artemisinin-based combination therapies (ACTs), which represent the gold-standard malaria treatment [7]. The failure rate of first-line ACT in treating *Plasmodium falciparum* infection was estimated to be 10% in the southeastern Asia region, which increased to 93% in Thailand [8]. Due to the emergence and rapid spread of multidrug-resistant *Plasmodium* parasites, there is an urgent need to discover novel agents to treat malaria.

For many centuries, numerous investigators have attempted to explore new potential and safe antimalarial agents to overcome the mechanisms of parasite resistance [9]. Plants and herbs have been a main source of drugs that are being developed to offer a potential treatment option for several diseases [10]. Antimalarial drugs such as quinine and artemisinin originate from plants and have been historically used as common malaria treatments [11, 12]; the antimalarial quinine is derived from the bark of *Cinchona rubra*, and artemisinin was discovered from *Artemisia annua* [13, 14]. Notably, antimalarial drugs have been developed from natural products and their derivatives. Therefore, natural plant materials are important sources of novel antimalarial agents.

*Spondias pinnata* (L.f.) Kurz (also known as *Spondias mangifera*) is in the Anacardiaceae family, and it is widely present in tropical, subtropical and temperate regions. This plant is a middle-sized evergreen tree with odd-pinnate compound leaves, polygamous flowers, elliptical-shaped fruits, and a pleasant aromatic acidic smell [15, 16]. Interestingly, parts of *S. pinnata*, such as the bark, roots, fruits, and leaves, have been used to treat various human diseases [17]. A root bark extract of *S. pinnata* has been applied to treat gonorrhea, diarrhea, dysentery, rheumatism, and severe cough [18, 19]. The bark extract of *S. pinnata* plays an important role as an antioxidant, free radical scavenger, and iron chelator [20, 21]. A leaf mash decoction of *S. pinnata* has been used to treat leprosy, whereas an inoculation of the leaves has been used to treat eye infection. Several extracts of *S. pinnata* have been found to exhibit antibacterial effects against *Helicobacter pylori* and *Salmonella typhymurium* and to present antimycobacterial activity [22]. Recently, ethanolic leaf extracts of *S. pinnata* have been shown to have antimalarial activity against *Plasmodium berghei* infection [17].

According to our in vitro study, an ethanolic seed extract of *S. pinnata* possesses strong antimalarial activity (50% inhibitory concentration (IC_50_): 2.46 ± 0.69 μg/ml) and is weakly toxic to Vero cells (50% cytotoxic concentration (CC_50_): 48.03 ± 0.04 μg/ml). *S. pinnata* seeds are herbal plant constituents in the Mahanil-Tang-Thong formulation, which is used for antipyretic treatment in Thailand. Based on a literature search, there are no documents reporting the in vivo antimalarial activity of ethanolic seed extracts of *S. pinnata*. Therefore, the aim of this study was to evaluate the antimalarial activity of an ethanolic seed extract of *S. pinnata* against the *P. berghei* ANKA strain and its acute toxicity in mice.

## Materials and methods

### Plant material and extraction procedure

Dried *S. pinnata* seeds were purchased from a traditional Thai drug store in Nakhon Si Thammarat Province, Thailand. The plant material was identified and authenticated by a botanist at the School of Pharmacy, Walailak University. A voucher of the plant sample (SMD 011018005) was deposited at the School of Medicine, Walailak University. The ethanolic extract was prepared at a 1:10 (w/v) ratio by the maceration method according to our previous report [8]. For the animal experiments, the plant extract was dissolved in 7% Tween 80 and 3% ethanol in distilled water.

### Phytochemical screening

Qualitative phytochemical screening for the identification of plant secondary metabolites in the ethanolic extract was performed according to standard methods, with some modifications [23–25]. Screening was performed based on coloration and precipitation reactions with specific reagents to evaluate the presence of flavonoids, terpenoids, alkaloids, tannins, anthraquinones, cardiac glycosides, saponins and coumarins.

### Test for flavonoids

A 5 ml ethanolic extract solution was heated and then filtered. A small piece of magnesium ribbon was added to the filtrate, and a few drops of concentrated HCl were then added. The formation of a pink, orange, or red-to-purple coloration indicated the presence of flavonoids.

### Test for terpenoids

A 5 ml ethanolic extract solution was mixed with 2 ml of chloroform, and 3 ml of concentrated H_2_SO_4_ was carefully added to form a layer. The formation of reddish-brown coloration at the interface indicated the presence of terpenoids.

### Test for alkaloids

A 5 ml ethanolic extract solution was mixed with 5 ml of 1% HCl. A few drops of Dragendorff’s reagent were added to the tube. The appearance of orange or orange-red precipitates indicated the presence of alkaloids.

### Test for tannins

A 0.5 g aliquot of dry extract was boiled in 5 ml of distilled water in a test tube and then filtered. A few drops of 1% ferric chloride solution were added to the filtrate. The appearance of brownish green or blue-black coloration indicated the presence of tannins.

### Test for anthraquinones

A 5 ml aliquot of extract solution was dried and shaken with 3 ml of petroleum ether. The filtrate was added to 2 ml of a 10% ammonia solution, and the mixture was shaken. The formation of red coloration indicated the presence of anthraquinones.

### Test for cardiac glycosides

A 5 ml aliquot of each extract solution was mixed with 2 ml of glacial acetic acid, and a few drops of 5% ferric chloride solution were added, followed by the addition of 1 ml of concentrated H_2_SO_4_. The formation of a brown ring at the interface indicated the presence of cardiac glycosides.

### Test for saponins

A 0.5 g aliquot of extract was dissolved in 5 ml of boiling water in a test tube and cooled. The filtrate was mixed with 3 ml of distilled water and shaken vigorously to create a stable persistent froth. The appearance of foam indicated the presence of saponins.

### Test for coumarins

Five milliliters of extract solution was dispensed into a test tube and covered with filter paper moistened with a 10% NaOH solution. The test tube was placed in boiling water for a few minutes, and then the filter paper was observed under UV light for yellow fluorescence. The appearance of greenish blue coloration indicated the presence of coumarins.

### GC–MS analysis

The GC–MS analysis of compounds in the ethanolic seed extract of *S. pinnata* was performed using an Agilent Technologies GC system with a 7000C GC/MS Triple Quad model (Agilent Technologies, Santa Clara, CA, USA) equipped with an HP-5MS column (30 m length × 0.25 mm diameter × 0.25 μm thickness). Spectroscopic detection by GC–MS involved an electron ionization system with a high ionization energy of 70 eV. The column temperature program was set as follows: 60 °C initially for 2 min and increased to 150 °C at 10 °C/min; finally, the temperature was increased to 300 °C at 5 °C/min. The carrier gas used was pure helium gas (99.99%) at a constant flow rate of 1 ml/min. The injector temperature was maintained at a constant temperature of 250 °C, and the solvent delay time was set to 2 min. A 1 μl sample was injected in split mode with a split ratio of 20:1. The identification of phytochemicals in the test samples was performed by comparing their mass spectra with the spectral database of known compounds in the National Institute of Standards and Technology (NIST) library. Only selected peaks with 80% similarity and above with NIST library entries were chosen and identified.

### Animals and rodent parasites

Twenty-six-day-old male ICR mice were obtained from Nomura Siam International Co., Ltd., Bangkok, Thailand. The animals were acclimatized for seven days in an environment-controlled room (12 h day-night light cycles, 22 ± 3 °C and 50–60% humidity) with food and clean water provided ad libitum. A rodent *P. berghei* ANKA strain was received from BEI Resources, NIAID, NIH, which was contributed by Thomas F. McCutchan. Mouse donors were infected intraperitoneally with *P. berghei*-infected cells. Blood was drawn from the heart when the mice had parasitemia levels of 20–30%.

### Animal grouping and dosing

For 4-day suppressive, curative, and prophylactic tests, the mice were randomly divided into six groups of five mice each. Group 1 (negative control group) was treated with solvent (a mixture of 7% Tween 80 and 3% ethanol in distilled water), and Group 2 (positive control group) was treated with 6 mg/kg body weight artesunate. Groups 3, 4, 5 and 6 (treatment groups) were treated with 250, 500, 600 and 800 mg/kg body weight extract, respectively. For acute toxicity testing, the mice were randomly divided into three groups of five mice each: Group 1 (untreated group) received no treatment; Group 2 (negative control group) received solvent (a mixture of 7% Tween 80 and 3% ethanol in distilled water); and Group 3 (treatment group) received a dose of 2,000 mg/kg body weight extract. Treatments were administered via oral gavage.

### Four-day suppressive test

The protocol was performed based on the Peters 4-day suppressive test [26]. All mice were injected intraperitoneally with 0.2 ml of 1 × 10^7^ infected red blood cells. Four hours after infection, the mice in each group were orally treated as described above and then continually treated for 3 days starting at 24 h after infection. On day five, blood was collected via the vascular tail vein and smeared on slides to prepare thin blood films. Each slide was stained with 10% Giemsa solution (Biotech Reagent Company Limited, Thailand). Parasitemia was determined under a light microscope (Olympus, model: CX-31, Japan) with an objective lens magnification power of 100x.

### Curative test

An assessment of the curative potential of the ethanolic *S. pinnata* seed extract was conducted according to a previous study [27]. Mice were infected with 0.2 ml of 1 × 10^7^ infected red blood cells via intraperitoneal injection. Afterward, the mice were orally treated once daily with their respective doses as described above, starting at 72 h post-infection, for 4 days. To monitor their parasitemia levels, Giemsa-stained thin blood film preparation was performed and assessed on day 7.

### Prophylactic test

The evaluation of the extract prophylactic activity was performed according to the method described by Ryley and Peters [27]. Both the control and experimental groups (five mice per group) were treated with solvent, drug or the extract as described in the animal grouping and dosing section for 3 consecutive days prior to infection. Twenty-four hours after receiving the last dose of treatment, the mice were injected with 0.2 ml of 1 × 10^7^ infected red blood cells via intraperitoneal injection. The parasitemia level was assessed at 72 h post-infection. Blood was taken from the vascular tail vein to perform thin blood film preparation and stained with Giemsa solution.

### Parasitemia suppression calculation

In vivo antimalarial activity tests were used to determine the effect of the extract against the parasite by calculating parasitemia using the following formula. Then, the percent parasitemia suppression was compared to that of the infected controls using the following formula:

$$\mathrm{\%Parasitemia }= \frac{\mathrm{Number of parasitized blood cells }}{\mathrm{Total red blood cells}}\times 100$$

$$\mathrm{\%Suppression }= \frac{\mathrm{Parasitemia }\left(\mathrm{negative control}\right)-\mathrm{Parasitemia }(\mathrm{treated group}) }{\mathrm{Parasitemia }\left(\mathrm{negative control}\right)}\times 100$$

### Mean survival time

In all of the in vivo antimalarial studies, mortality was monitored, and the number of days from the time of parasite inoculation death was recorded for each mouse in the treatment and control groups for 30 days (as an endpoint). At that point, any remaining mice were euthanized using an overdose of sodium pentobarbital (100 mg/kg). The mean survival time for each group was calculated as follows:

$$\mathrm{Mean survival time }= \frac{\mathrm{Sum of survival time of all mice in a group }(\mathrm{days}) }{\mathrm{Total number of mice in the group}}$$

### Acute oral toxicity

The acute toxicity of the ethanolic crude extract of *S. pinnata* seeds was determined in healthy mice according to standard guidelines [28]. Fifteen mice were randomly divided into three groups of five mice each as described in the animal grouping and dosing section. In brief, the mice in the treatment group were orally administered a single dose of 2,000 mg/kg body weight extract. The negative control group received a mixture of 7% Tween 80 and 3% ethanol in distilled water. Untreated mice served as the normal controls. The mice were observed for 3 h after treatment; thereafter, signs of toxicity or mortality were continually observed for 14 days. Food and water consumption was noted every day during the experimental period. Mouse body weight was measured using a sensitive digital weighing balance (Mettler Toledo, model: ML3002E, Indonesia) before treatment and after the completion of testing. At the end of this study, the mice were anesthetized by intraperitoneal injection of sodium pentobarbital (50 mg/kg body weight). After anesthetization, mouse blood was collected via cardiac puncture for biochemical analysis of liver and kidney functions, including aspartate aminotransferase (AST), alanine aminotransferase (ALT), alkaline phosphatase (ALP), blood urea nitrogen (BUN) and creatinine levels, with an AU480 chemistry analyzer (Beckman Coulter, USA). In addition, histological changes in liver and kidney tissues were evaluated using hematoxylin and eosin (H&E) staining.

### Histopathological evaluation

A histological study of the liver and kidney tissues was performed according to our previous study [8]. In brief, the tissues were fixed in a 10% formalin solution after removal from the mice. The fixed tissues were dehydrated by a series of increasing alcohol concentrations, diaphanized with xylene and embedded in paraffin. The samples were cut to obtain cross-sections measuring 5 µm thick. The sections were dewaxed, rehydrated and stained with hematoxylin–eosin. The histological changes were evaluated under a light microscope by an expert pathologist.

### Statistical analysis

The data were analyzed using SPSS for Microsoft Windows version 17.0 (SPSS, IL, USA). One-way ANOVA was performed followed by post hoc Tukey**’**s multiple comparison test. The results are expressed as the mean plus or minus the standard derivation **(**mean ± SD**)**. The level of significance was set at *P* < 0.05.

## Results

### Extractive value and phytochemical screening

The extractive yield for ethanolic seed extracts of *S. pinnata* was 3.18%. Phytochemical screening revealed the presence of terpenoids, tannins and coumarins (Table 1).

**Table 1:** Phytochemical screening of the ethanolic *S. pinnata* seed extract

Phytochemical constituents	Result
Flavonoids	**-**
Terpenoids	** + **
Alkaloids	**-**
Tannins	** + **
Anthraquinones	**-**
Cardiac glycosides	**-**
Saponins	**-**
Coumarins	** + **

( +): presence, (-): absence of phytochemical constituents

### GC–MS analysis

**Fig. 1:**
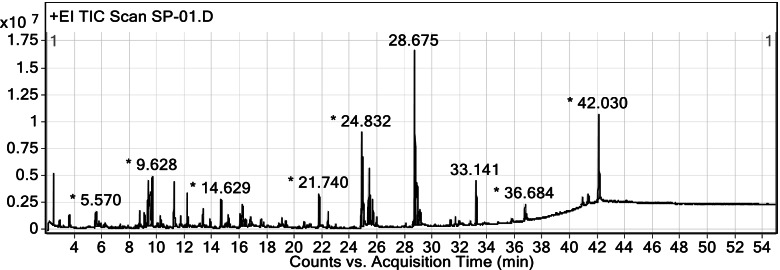
GC–MS chromatogram of ethanolic *S. pinnata* seed extract

The GC–MS chromatograms of the ethanolic *S. pinnata* seed extract are illustrated in Fig. 1. The mass spectrum of phytochemical compounds was compared with the spectral database of known compounds of the NIST library. Twenty-nine compounds were identified and characterized, as listed in Table 2. The most abundant compound was oleic acid amide (11.02%) with a retention time of 28.675 min, followed by β-sitosterol (6.45%), linoleic acid (5.41%), and oleic acid (5.37%). Other compounds were present at less than 5%, such as linoleic acid ethyl ester, palmitic acid, vanillin, protocatechuic acid, syringic acid and gallic acid.

**Table 2:** Compounds identified in the ethanolic *S. pinnata* seed extract by GC–MS

No	RT (min)	Compound name	Molecular formula	MW	Peak area (%)
1	2.472	Acetal	C_6_H_14_O_2_	118	2.02
2	3.609	Furfural	C_5_H_4_O_2_	96	0.50
3	9.628	5-Hydroxymethylfurfural	C_6_H_6_O_3_	126	1.75
4	12.162	Vanillin	C_8_H_8_O_3_	152	1.34
5	13.327	Levoglucosan	C_6_H_10_O_5_	162	1.52
6	13.839	Phenol,2,4-bis(1,1-dimethylethyl)-	C_14_H_22_O	206	0.35
7	14.629	3-Hydroxy-4-methoxybenzoic acid	C_8_H_8_O_4_	168	1.58
8	17.529	Protocatechuic acid	C_7_H_6_O_4_	154	0.59
9	19.061	Syringic acid	C_9_H_10_O_5_	198	0.50
10	20.305	Dodecanoic acid, 3-hydroxy-	C_12_H_24_O_3_	216	0.10
11	21.078	Gallic acid	C_7_H_6_O_5_	170	0.33
12	21.740	Palmitic acid	C_16_H_32_O_2_	256	2.04
13	22.392	Ethyl palmitate	C_18_H_36_O_2_	284	0.83
14	22.944	Isopropyl palmitate	C_19_H_38_O_2_	298	0.18
15	24.832	Linoleic acid	C_18_H_32_O_2_	280	5.41
16	24.918	Oleic acid	C_18_H_34_O_2_	282	5.37
17	25.304	Octadecanoic acid	C_18_H_36_O_2_	284	1.21
18	25.362	Linoleic acid ethyl ester	C_20_H_36_O_2_	308	3.21
19	25.460	Ethyl oleate	C_20_H_38_O_2_	310	1.64
20	25.632	Palmitic acid amide	C_16_H_33_NO	255	1.43
21	25.908	Ethyl stearate	C_20_H_40_O_2_	312	0.43
22	28.580	E,E,Z-1,3,12-Nonadecatriene-5,14-diol	C_19_H_34_O_2_	294	0.24
23	28.675	Oleic acid amide	C_18_H_35_NO	281	11.02
24	28.975	Spiculesporic acid	C_17_H_28_O_6_	328	1.26
25	29.080	Octadecanamide	C_18_H_37_NO	283	1.66
26	31.280	Benzophenone-6	C_15_H_14_O_5_	274	0.37
27	31.909	cis-11-Eicosenamide	C_20_H_39_NO	309	0.38
28	33.141	10,11-Dihydro-10-hydroxy-2,3-dimethoxydibenz(b,f)oxepin	C_16_H_16_O_4_	272	2.58
29	42.030	β-Sitosterol	C_29_H_50_O	414	6.45

*RT* Retention time

### Four-day suppressive test

The in vivo antimalarial properties of ethanolic *S. pinnata* seed extract against the *P. berghei* ANKA strain were examined by using a 4-day suppressive test. Mice treated with 250, 500, 600, and 800 mg/kg doses of the extract showed a significant percentage suppression of parasitemia in a dose-dependent manner, with 22.94, 49.01, 60.67 and 66.82%, respectively, compared to that of the negative control group (*P* < 0.05). Mice in the positive control group showed a significantly greater percentage suppression of parasitemia than the mice in all the treatment groups (*P* < 0.05) (Table 3). With regard to the mean survival time, the ethanolic seed extract also prolonged the survival time of the treatment group mice at doses of 250, 500, 600, and 800 mg/kg. The mice in the 800 mg/kg treatment group showed the highest mean survival time of 16.20 ± 2.03 days and had a significantly increased mean survival time compared with that of the negative control group mice (*P* < 0.05) (Table 3). Interestingly, the mean survival time of the mice treated with 800 mg/kg extract was comparable to that of the positive control group mice (18.40 ± 0.88 days).

**Table 3:** Effect of the ethanolic *S. pinnata* seed extract on parasite level, parasite suppression, and mean survival time during the 4-day suppressive test

Group	Dose (mg/kg)	% Parasitemia	% Suppression	Mean survival time (days)
Negative control	-	33.44 ± 0.18^b,c,d,e,f^	-	9.60 ± 0.61^b,f^
Positive control	6	1.10 ± 0.21^a^^,^^c^^,d,e,f^	96.72 ± 0.62^a^^,^^c^^,d,e,f^	18.40 ± 0.88^a^^,^^c^^,d^
*S. pinnata* extract	250	25.77 ± 0.34^a^^,^^b^^,d,e,f^	22.94 ± 1.02^a^^,^^b^^,d,e,f^	10.20 ± 0.92^a^^,f^
	500	17.05 ± 1.04^a^^,^^b^^,^^c^^,e,f^	49.01 ± 3.10^a^^,^^b^^,^^c^^,e,f^	13.20 ± 1.99^b^
	600	13.15 ± 1.11^a^^,^^b^^,^^c^^,d,f^	60.67 ± 3.32^a^^,^^b^^,^^c^^,d,f^	14.00 ± 2.05
	800	11.10 ± 1.34^a^^,^^b^^,^^c^^,d,e^	66.82 ± 4.00^a^^,^^b^^,^^c^^,d,e^	16.20 ± 2.03^a^^,^^c^

Data are presented as the means ± SDs (*n* = 5 per group)

^a^Compared with the negative control group receiving a mixture of 7% Tween 80 and 3% ethanol in distilled water, ^b^compared with the positive control group receiving 6 mg/kg artesunate, ^c^compared with 250 mg/kg extract, ^d^compared with 500 mg/kg extract, ^e^compared with 600 mg/kg extract, and ^f^compared with 800 mg/kg extract

### Curative activity test

The results of the curative activity testing for the ethanolic *S. pinnata* seed extract are shown in Table 4. Mice treated with 250, 500, 600 and 800 mg/kg doses of the extract exhibited a significant percentage suppression of parasitemia, at 17.05, 42.46, 56.79, and 51.54%, respectively, compared to that of the negative control group mice. However, the ethanolic *S. pinnata* seed extract displayed lower curative activity than the standard drug artesunate. No significant difference in mean survival time was observed in any treatment group or the positive control group when compared with that of the negative control group.

**Table 4:** Effect of the ethanolic *S. pinnata* seed extract on the parasite level and parasite suppression in the curative activity test

Group	Dose (mg/kg)	% Parasitemia	% Suppression	Mean survival time (days)
Negative control	-	32.94 ± 2.98^b,c,d,e,f^	-	10.00 ± 3.29
Positive control	6	4.71 ± 0.92^a^^,^^c^^,^^d^^,^^e^^,^^f^	85.71 ± 2.79^a^^,^^c^^,^^d^^,^^e^^,^^f^	16.60 ± 1.85^c,d^
*S. pinnata* extract	250	27.32 ± 1.14^a^^,^^b^^,^^d^^,^^e^^,^^f^	17.05 ± 3.47^a^^,^^b^^,^^d^^,^^e^^,^^f^	7.20 ± 1.10^b^
	500	18.96 ± 1.19^a^^,^^b^^,^^c^^,^^e^^,^^f^	42.46 ± 3.62^a^^,^^b^^,^^c^^,^^e^^,^^f^	8.20 ± 1.30^b^
	600	14.23 ± 1.22^a^^,^^b^^,^^c^^,^^d^	56.79 ± 3.70^a^^,^^b^^,^^c^^,^^d^	9.60 ± 1.14
	800	15.96 ± 1.10^a^^,^^b^^,^^c^^,^^d^	51.54 ± 3.33^a^^,^^b^^,^^c^^,^^d^	13.20 ± 6.98

Data are presented as the means ± SDs (*n* = 5 per group)

^a^Compared with the negative control group receiving a mixture of 7% Tween 80 and 3% ethanol in distilled water, ^b^compared with the positive control group receiving 6 mg/kg artesunate, ^c^compared with 250 mg/kg extract, ^d^compared with 500 mg/kg extract, ^e^compared with 600 mg/kg extract, and ^f^compared with 800 mg/kg extract

### Prophylactic activity test

Mice treated with doses of 250, 500, 600, and 800 mg/kg showed a significant percentage suppression of parasitemia, at 13.35, 24.99, 25.49, and 31.06%, respectively, when compared to that of the negative control group mice (Table 5). Mice treated with 6 mg/kg artesunate (positive control group) showed 33.93% suppression of *P. berghei* parasitemia. An interesting result was observed at 800 mg/kg, when the suppression of parasite levels (31.06%) was comparable to that of the standard drug. In addition, the ethanolic seed extract increased the mean survival time in a dose-dependent manner. However, there was no significant difference in the mean survival time among the negative control, positive control, and treatment groups.

**Table 5:** Effect of the ethanolic *S. pinnata* seed extract on parasite levels and parasite suppression during the prophylactic test

Group	Dose (mg/kg)	% Parasitemia	% Suppression	Mean survival time (days)
Negative control	-	20.39 ± 0.96^b,c,d,e,f^	-	11.60 ± 4.59
Positive control	6	13.47 ± 0.62^a^^,^^c^^,^^d^^,^^e^	33.93 ± 3.03^a^^,^^c^^,^^d^^,^^e^	16.20 ± 4.79
*S. pinnata* extract	250	17.67 ± 0.81^a^^,^^b^^,^^d^^,^^e^^,^^f^	13.35 ± 3.99^a^^,^^b^^,^^d^^,^^e^^,^^f^	11.40 ± 1.52
	500	15.29 ± 1.03^a^^,^^b^^,^^c^^,^^f^	24.99 ± 5.05^a^^,^^b^^,^^c^^,^^f^	14.60 ± 3.91
	600	15.19 ± 0.53^a^^,^^b^^,^^c^^,^^f^	25.49 ± 2.58^a^^,^^b^^,^^c^^,^^f^	14.40 ± 4.04
	800	14.06 ± 0.79^a^^,^^c^^,^^d^^,^^e^	31.06 ± 3.87^a^^,^^c^^,^^d^^,e^	15.20 ± 3.90

Data are presented as the means ± SDs (*n* = 5 per group)

^a^Compared with the negative control group receiving a mixture of 7% Tween 80 and 3% ethanol in distilled water, ^b^compared with the positive control group receiving 6 mg/kg artesunate, ^c^compared with 250 mg/kg extract, ^d^compared with 500 mg/kg extract, ^e^compared with 600 mg/kg extract, and ^f^compared with 800 mg/kg extract

### Acute oral toxicity test

#### General health and behavior, food and water consumption, and body weight

Mice treated with a single dose of 2,000 mg/kg body weight ethanolic seed extract did not exhibit mortality or major behavioral alterations, such as hair erection, appetite loss, vomiting, diarrhea, secretion changes, abnormal sleep patterns and tremors. Regarding the effect of *S. pinnata* seed extract on food and water consumption during the acute toxicity test, the mean water and food consumption of the mice in the treatment group with a single dose of 2,000 mg/kg extract and those in the negative control group showed no statistically significant difference when compared with that of the mice in the untreated control group (*P* > 0.05) at week 1 and week 2 (Table 6). In addition, the mean percent changes in body weight for the mice in the treatment group receiving 2,000 mg/kg extract and the negative control group were not significantly different when compared with those of the mice in the untreated control group (*P* > 0.05) (Table 7).

**Table 6:** Effect of the ethanolic *S. pinnata* seed extract on mouse food and water consumption in the acute toxicity test

Food consumption (g)	Untreated control	Negative control	2,000 mg/kg extract
Week 1	26.29 ± 3.15	27.29 ± 2.36	26.00 ± 2.24
Week 2	25.71 ± 2.14	26.86 ± 1.95	24.29 ± 3.68
Water consumption (mL)			
Week 1	49.71 ± 3.04	50.57 ± 1.72	51.71 ± 2.36
Week 2	69.86 ± 3.02	68.57 ± 8.16	70.00 ± 4.62

Data are presented as the means ± SDs (*n* = 5 per group)

**Table 7:** Differences in body weight in the acute toxicity test before (D0) and 14 days after (D14) administration of the ethanolic *S. pinnata* seed extract

Group	Mean body weight (g)		
	**D0**	**D14**	**% Change**
Untreated control	34.16 ± 1.28^b^	39.84 ± 2.16	14.17 ± 1.93
Negative control	34.38 ± 0.63^b^	40.57 ± 0.64	15.26 ± 0.82
2,000 mg/kg extract	32.55 ± 0.90^a^	38.35 ± 2.47	14.96 ± 3.81

Data are presented as the means ± SDs (*n* = 5 per group)

^a^Compared with the negative control group that received a mixture of 7% Tween 80 and 3% ethanol in distilled water and ^b^compared to the treatment group that received a dose of 2,000 mg/kg body weight

#### Kidney and liver functions

The biochemical parameters for liver function, including the AST, ALT, and ALP levels, in the mice treated with a single 2,000 mg/kg dose were not significantly different from those in the mice in the untreated control and negative control groups (*P* > 0.05) (Table 8). Regarding the kidney function biochemical parameters, the level of creatinine in the mice treated with a single 2,000 mg/kg dose exhibited no significant difference from that in mice from the untreated control and negative control groups (*P* > 0.05). The BUN level in the mice treated with a single 2,000 mg/kg dose was less than that in the mice from the untreated control group and significantly decreased compared with that in the mice from the negative control group (*P* < 0.05) (Table 8).

**Table 8:** Effect of ethanolic *S. pinnata* seed extract on kidney and liver functions in the acute toxicity test

Parameter	Untreated control	Negative control	2,000 mg/kg extract
**Liver function test**			
AST (U/L)	86.80 ± 5.15	87.50 ± 2.50	83.40 ± 5.64
ALT (U/L)	35.36 ± 3.30	37.50 ± 3.20	36.20 ± 4.02
ALP (U/L)	85.90 ± 4.09	86.03 ± 3.23	83.40 ± 2.07
**Kidney function test**			
BUN (mg/dL)	25.36 ± 2.88	27.72 ± 1.30^b^	24.36 ± 0.68^a^
Creatinine (mg/dL)	0.64 ± 0.03	0.66 ± 0.04	0.63 ± 0.02

Data are presented as the means ± SDs (*n* = 5 per group)

^a^compared to the negative control group, and ^b^compared to the treatment group that received a dose of 2,000 mg/kg body weight

#### Histopathological examination of the kidney and liver tissues

**Fig. 2:**
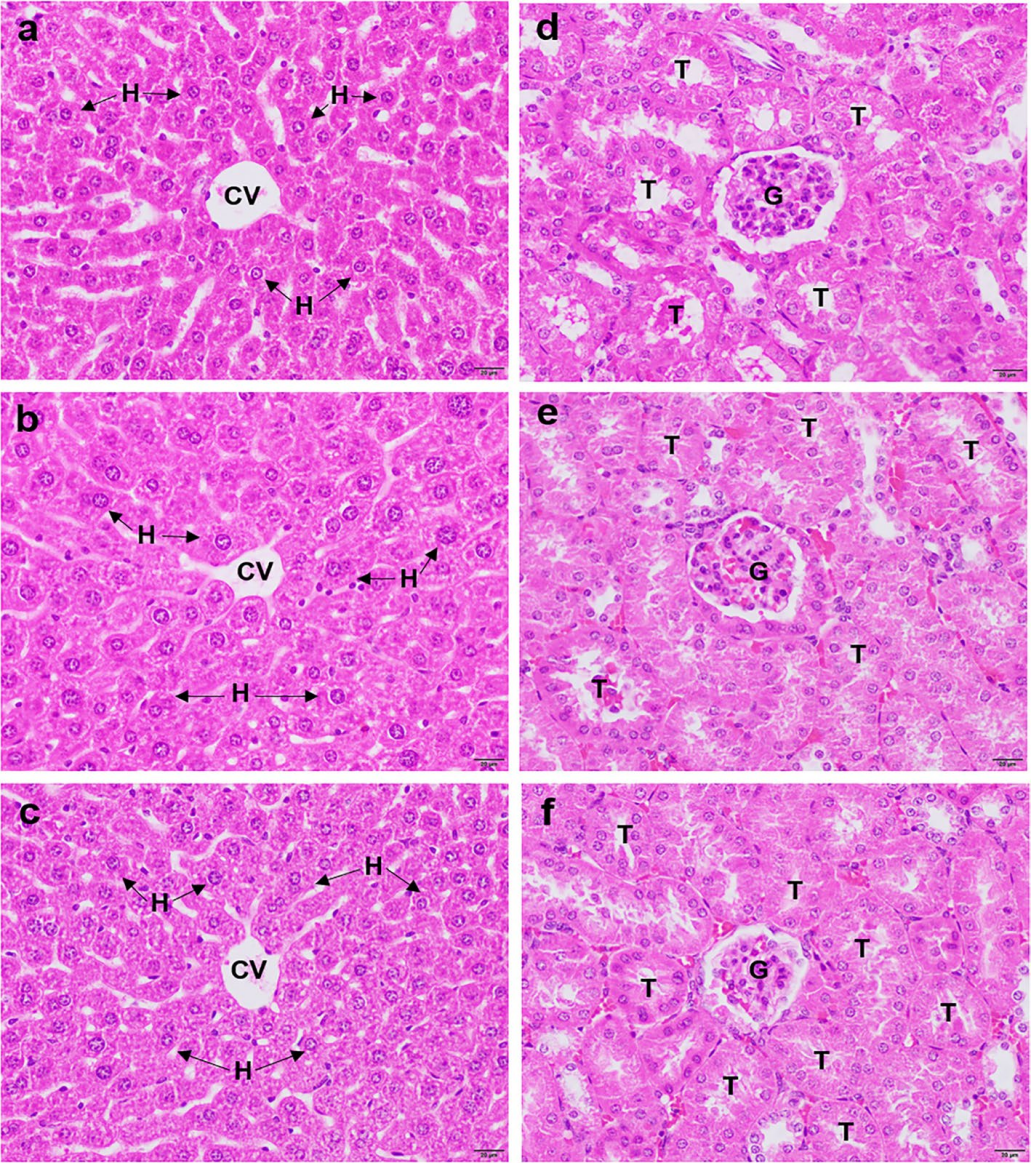
Histopathological changes in liver and kidney tissues from the untreated control group (**a**) and (**d**), negative control group (**b**) and (**e**), and treatment group that received a single dose of 2,000 mg/kg body weight (**c**) and (**f**). All images were acquired at 20X magnification. Bar = 20 μm. Central vein (CV), hepatocyte (H), tubule (T), and glomerulus (G)

The histopathological findings for the kidney and liver tissues are shown in Fig. 2. The liver tissues from the mice treated with a single 2,000 mg/kg dose revealed normal hepatocyte morphology with a pink cytoplasm and normal structures for the hepatic sinusoids and the central vein. No evidence of sinusoid vasodilation or inflammatory cell infiltration (Fig. 2E) was observed when compared with the tissues from the untreated control (Fig. 2A) and the negative control groups (Fig. 2C). In addition, the kidney tissue from the mice treated with a single dose of 2,000 mg/kg *S. pinnata* extract demonstrated normal histology and glomerulus, Bowman’s capsule, and kidney epithelial cell structures (Fig. 2F) when compared to those of the untreated control (Fig. 2B) and the negative control group tissues (Fig. 2D).

## Discussion

Because natural products are sources of active compounds, numerous researchers have designed tests to evaluate new substances for resolving drug resistance problems. This study aims to stimulate novel antimalarial development. Our in vitro study showed that the ethanolic *S. pinnata* seed extract had antimalarial activity against *P. falciparum*, with an IC_50_ value of 2.46 ± 0.69 μg/ml, which was categorized as strong malarial activity [8]. A selectivity index (SI) greater than ten represents a favorable safety window, which is determined from the ratio between the toxic concentration to human cells (CC_50_) and the effective concentration to inhibit the parasite (IC_50_) [29]. Our in vitro study demonstrated that the ethanolic *S. pinnata* seed extract had an SI greater than ten. Therefore, the ethanolic seed extract of S*. pinnata* exhibited promising optimum selective antimalarial activity for further in vivo study because it exhibits potent in vitro therapeutic effects with low toxicity. In the present animal model study, ICR mice were selected for the investigation of antimalarial activity because they are very susceptible to *P. berghei* ANKA infection. Regarding *P. berghei* ANKA, this strain is the parasite of choice for these assays because of its ability to sequester within the microcirculation, which is characteristic of severe malaria [30]. Three methods were applied to evaluate the antimalarial activity of the ethanolic *S. pinnata* seed extract. The 4-day suppressive test is the standard test for early infections and is primarily used for antimalarial drug screening. The curative test evaluates the curative capability of extracts against established infections. Additionally, a prophylactic test that addresses the prophylactic activity of extracts is also a common test for assessing antimalarial activity [31]. In all these methods, the most reliable parameter is the percent suppression of parasitemia [32]. However, the mean survival time, which is also an important parameter of an antimalarial assay, was also determined.

In the present study, the 4-day suppressive test showed parasitemia suppression in a dose-dependent manner, with the highest percentage in mice treated with 800 mg/kg extract (66.82%). The ethanolic *S. pinnata* seed extract also provided a significant extension of the survival time in the treatment group compared to the negative control group. This prolonged survival time of the infected mice could be due to the suppression of parasitemia and a reduction in the overall pathologic effect [32]. These findings indicated that ethanolic *S. pinnata* seed extract at the 800 mg/kg dose could be useful for suppressing early malaria infection. The curative property against established infection was found to reach maximum suppression at a dose of 600 mg/kg. This result may imply that the ethanolic seed extract did not produce greater activity at higher doses.

The suppression and curative activities were more potent than the prophylactic activity, which might be due to poor bioavailability, fast elimination or other pharmacokinetic and pharmacodynamic properties of the extract because the extract was administered before infection during the prophylactic test. However, prolonging the survival time beyond 12 days is an important index for evaluating the antimalarial activity of plant extracts. Mice that received an 800 mg/kg dose of the extract exhibited the longest mean survival times beyond 12 days, which indicates that the extract was active [12]. Chemicals produced by plants known as phytochemicals have provided protective health benefits. In our study, a phytochemical analysis of the ethanolic *S. pinnata* seed extract revealed the presence of terpenoids, tannins, and coumarins. These constituents may act as active metabolites against the parasite. Terpenoids are derived from five-carbon isoprene units [33]. In pharmaceuticals, terpenoids have many potential properties, including antioxidant, antiaging, anti-inflammatory, antibacterial, antiviral, antimalarial, and neuroprotective effects [34]. Moreover, terpene compounds from medicinal plants have been reported to inhibit heme crystallization, leading to parasite death [35]. Tannins are diverse phenolic compounds that are able to inactivate and kill microorganisms, and they have been revealed to have anti-inflammatory, antiseptic, antioxidant and antiplasmodial properties [36, 37]. Coumarins are natural substances found in many plants, and they play an important role in medicinal applications such as antibiotic, antimitotic, antiviral, anticancer, anti-inflammatory, anticoagulant, antifungal, antioxidant and immunomodulatory activities [38].

Because secondary metabolites have shown several biological effects, the antimalarial activity of *S. pinnata* may proceed through many possible mechanisms, such as antihemozoin effects from terpenoids, DNA synthesis inhibition by coumarins, antioxidant effects to inhibit heme polymerization or other unknown mechanisms [35, 39, 40]. Therefore, the antimalarial activity of the *S. pinnata* extract may have been influenced by a single phytoconstituent or a combination of the mentioned phytoconstituents in the crude extracts.

The GC–MS analysis of ethanolic *S. pinnata* seed extracts revealed the presence of various compounds. The major compounds were oleic acid amide, β-sitosterol, linoleic acid, and oleic acid, and they included linoleic acid ethyl ester, palmitic acid, vanillin, protocatechuic acid, syringic acid and gallic acid. Some of the constituents revealed by GC–MS are biologically active compounds. Linoleic acid has been shown to possess antioxidant, anti-glycemic and hypolipidemic activities [41]. β-Sitosterol has been reported to show high activity against chloroquine-sensitive (3D7) strains, with an IC_50_ value of 5.51 µM [42]. Vanillin has been shown to exhibit anticancer, antiangiogenic, analgesic, anti-inflammatory, antifungal, antibacterial and antiviral effects [43, 44]. Protocatechuic acid is well known to exhibit antioxidant, anti-inflammatory, antihyperglycemic, antibacterial, anticancer, and antispasmodic properties [45]. This compound has been confirmed to have moderate antimalarial activity [46]. Gallic acid has several pharmacological effects, including antibacterial, antidiabetic, antitumor, antiobesity, and antiplasmodial activities [47, 48]. Therefore, the antimalarial activity of the *S. pinnata* extract may be produced by a single phytoconstituent or the synergistic effect of these compounds, as mentioned above.

According to the acute oral toxicity test, the ethanolic seed extract did not reveal any signs of toxicity or lethality during the 14 days. Therefore, the oral lethal dose 50% (LD_50_) value should be greater than 2,000 mg/kg. As a result, according to the OECD’s Globally Harmonized System of Classification [28], the ethanolic *S. pinnata* seed extract can be classified as category 5 with a relatively low acute toxicity hazard. In this study, food and water consumption and body weight changes were also measured to monitor the toxicity of the *S. pinnata* extract since these parameters can also be used to indicate the harmful effects of extracts [49]. Food and water consumption were not significantly different between the untreated control and negative control groups. There was no significant difference in weight among all groups, which may indicate that the extract does not affect metabolism in the animals.

In fact, the liver and kidney are important targets of toxic substances [50]. This study demonstrated that the biochemical parameters of liver function, including the AST, ALT and ALP levels, were not significantly changed in the mice treated with a single 2,000 mg/kg body weight dose. In addition, no histopathological changes were observed in the liver or kidney tissues. These findings indicated no acute toxic effect on mice at a dose of 2,000 mg/kg. Therefore, notable antimalarial activity with low extract toxicity must be confirmed, and determine the detailed structures of the pure compounds must be investigated in further investigations.

## Conclusion

The *S. pinnata* seed extract contains various phytochemical compounds with important medicinal properties. This study was the first to demonstrate that the ethanolic *S. pinnata* seed extract possessed promising antimalarial activity against *P. berghei* ANKA and had no toxicity. The extract showed a significant percentage suppression of parasitemia in a dose-dependent manner, prolonged the mean survival time and exhibited significant curative and prophylactic activities. The findings from the present study provide scientific evidence supporting the use of *S. pinnata* seeds in developing new drugs for malaria treatment. This study has limitations that will be investigated in future studies. Although GC–MS analysis was performed to characterize the compounds in the *S. pinnata* seed extract, the active antimalarial compounds were not identified. Additional studies are needed to isolate and identify the active compounds, as well as to understand the mechanism of inhibition.

## Data Availability

The data associated with this study are included in this published article. Additional files are available from the corresponding author upon reasonable request.
